# The Influence of the Plant Biomass Pyrolysis Conditions on the Structure of Biochars and Sorption Properties

**DOI:** 10.3390/molecules30142926

**Published:** 2025-07-10

**Authors:** Bernadetta Kaźmierczak, Jolanta Drabik, Paweł Radulski, Anna Kaczmarczyk, Edyta Osuch-Słomka

**Affiliations:** 1 Bioeconomy and Ecoinnovation Centre, Łukasiewicz Research Network—Institute for Sustainable Technologies, 26-600 Radom, Poland; jolanta.drabik@itee.lukasiewicz.gov.pl (J.D.); pawel.radulski@itee.lukasiewicz.gov.pl (P.R.); anna.kaczmarczyk@itee.lukasiewicz.gov.pl (A.K.); 2 Tribology Center, Łukasiewicz Research Network—Institute for Sustainable Technologies, 26-600 Radom, Poland; edyta.slomka@itee.lukasiewicz.gov.pl

**Keywords:** plant biomass, pyrolysis, corn biochars, apple biochars, FTIR, Raman

## Abstract

The aim of this work was to obtain biochar materials from plant biomass and to determine the changes occurring under the conditions of the pyrolysis process and physical activation, as well as to characterize the physicochemical characteristics of the produced products in terms of their practical use. The pyrolysis process was carried out at a temperature of 700 °C, under the flow of a protective gas, i.e., carbon dioxide, at a rate of 5.0 L/min. The pyrolysis processes were carried out in the absence and presence of an activating agent. For ecological safety, physical activation using water vapor was chosen. In the next stage of the work, biochars were produced and subjected to detailed physicochemical analysis. A scanning electron microscope with energy-dispersive SEM/EDS was used to determine the microstructure and changes in the chemical composition of the biochars. FTIR spectrophotometry was used to identify the functional groups present in the structures of biochars and to indicate changes occurring in the biomass during pyrolysis. Meanwhile, Raman spectroscopy was used to assess the ordering of the biochar structures based on the identification of spectral signals. The description of the specific surface areas of the biochars was made possible by studies conducted using a physical and chemical adsorption analyzer. Based on the obtained research results, the elementary structure, surface development, presence of functional groups on the surfaces of biochars and changes in the structure before and after activation with water vapor were determined. It was found that the biochars had functional groups, a well-developed specific surface area that increased after activation with water vapor, micropores and mesopores, as well as changes in structure under the influence of physical activation. It has been shown that the presence of functional groups influences the hydrogen sulfide sorption capacity.

## 1. Introduction

The intensive development of the agri-food and fruit and vegetable industries, as well as the global growth of agriculture, leads to the generation of waste residues [1]. Most of the waste generated in agriculture is incinerated, which results in the emission of pollutants into the atmosphere. This approach to waste has been a serious problem in recent years [1]. In Poland, approximately 5 million tons of biodegradable waste is produced from the agri-food and fruit and vegetable processing sectors [2]. A large part of this is not processed and constitutes waste that can be managed, and, through appropriate processes, a new utility product can be created that can be reused [3]. The current European Union regulations regarding the principles of sustainable development, the Green Deal and the circular economy require the development of biodegradable and environmentally friendly products. One type of waste from which new materials with a wide range of uses can be obtained is biomass [3]. According to the definition included in the European Union regulations (Directive 2018) [4], as well as in the Polish legislation (Journal of Laws 2018, item 1276) [5], biomass may consist of corn waste (including stalks, leaves and other residues) or apple waste (including pomace) [3,6]. The plant biomass from corn and apples can be a raw material for the production of heat energy and biogas, but also for the production of other products, such as biochars [7,8,9]. The main components of corn biomass are C—45.7%, O—41.1%, H—5.3% and N—0.7% [9], whereas that of apples contains C—47.1%, O—45.9%, H—6.4% and N—0.5% [6]. The presence of these elements in their structures makes it possible to shape the chemical structures and active surfaces of biochars during their production [10,11,12].

Biochars, due to their wide range of applications, can be used as sorbents for water treatment or in the purification of wastewater from metals or gases. The chemical composition of industrial wastewater is not constant; even after the same process carried out in the plant, it may change. For this reason, it is difficult to choose appropriate methods for their purification. Thus, we are constantly seeking methods to resolve this problem. One such method may be adsorption on carbon materials. Activated carbon is commonly used in the adsorption process. Activated carbons can be used to remove organic pollutants from water and sewage, e.g., petroleum derivatives, polycyclic aromatic hydrocarbons (PAHs), phenols, detergents, metals or gaseous compounds, including sulfur compounds [3,13,14]. Sulfur compounds, including hydrogen sulfide in water and sewage, are responsible for their unpleasant odor. Currently, research results appear in the literature [3,14,15,16,17,18,19,20,21] that demonstrate the possibility of using biochars for the adsorption of inorganic pollutants.

Our own research described in [3] allowed us to obtain information on the influence of the elementary structure and surface development of biochar sorbents on the efficiency of chromium (III) removal from water solutions using biochars obtained from corn at a temperature of 500 °C. The analyses performed showed the presence of organooxygen functional groups on the surfaces of biochars, which showed sorption properties towards chromium (III). Tan et al. [16] used biochars obtained from sewage sludge for the adsorption of chromium (III) from water solutions. The produced biochars enabled the adsorption of chromium (III) at the level of 80% due to the presence of oxygen groups (–OH) on the surfaces of the biochars. According to Tyagi [17], biochars obtained from plant biomass are not only capable of adsorbing chromium but also arsenic, copper, nickel, cadmium and lead. Hashem et al. [18] indicated that the adsorption of chromium from aqueous solutions can be achieved using biochars obtained from pumpkin peels. The obtained biochars had functional groups on their surfaces, capable of adsorption. The studies described by Khazaei et al. [22] showed that it is possible to use biochars obtained from almond and apricot shells for the adsorption of heavy metals from water solutions. Biochars showed the presence of functional groups such as O–CH3, C=O, C–H and O–H on their surfaces, which participate in metal adsorption. The studies conducted by Rodrigo et al. [23] showed that it is possible to use biochars obtained from pine biomass in the adsorption of Pb (II) and Cd (II) from water solutions. The carried out studies showed the presence of functional groups on the surfaces of biochars, such as C=O, O–H, C–O and –COOH, which participated in the adsorption of Pb (II) and Cd (II). Similar results were obtained by Yuan et al. [24]. Biochars obtained from cotton stems additionally modified with polyethylene showed sorption capacity for Pb (II). The obtained biochars had functional groups on their surfaces, such as C=O and O–H, which participated in Pb (II) adsorption. Chen et al. [25], in their study, used biochars obtained from lettuce that were additionally modified with H_3_PO_4_ for the adsorption of Cd (II) from aqueous solutions. The conducted studies showed the presence of functional groups on the biochar, participating in adsorption.

Biochars obtained during the pyrolysis of plant waste may be characterized by a complex chemical structure and contain numerous organooxygen functional groups, the occurrence and chemical qualities of which depend on the process conditions—in particular, on the pyrolysis rate and temperature [10,11,12]. The pyrolysis process carried out at a temperature of 500 °C influences the formation of oxygen functional groups on the surfaces of biochars [11,12,26]. According to our own research [3], biochars obtained from corn at a temperature of 500 °C have, on their surfaces, oxygen organic functional groups such as carboxyl, carbonyl and hydroxyl. According to the research described in [11], biochars obtained from tea waste at a temperature of 500 °C had oxygen organic functional groups, mainly carbonyl groups. On the other hand, increasing the pyrolysis temperature above 500 °C causes a decrease in the content of surface functional groups containing oxygen and hydrogen [11,14,27]. According to the studies described in [11], biochars obtained at 700 °C from corn and tea waste show the slight disappearance of the bands indicating the presence of organooxygen groups. Similar research results were observed in [14].

In addition to the temperature, the type of activation has a significant impact on the properties of biochars. There are two activation methods—chemical and physical—which differ in terms of the mechanism, type of activating agent used and physicochemical properties of the obtained products [28]. Chemical activation involves the introduction of additives such as zinc chloride, phosphoric acid, potassium hydroxide and ammonium salts during carbonization at a temperature of 300–500 °C [29]. For example, the modification of biochars with H_3_PO_4_ causes an increase in the active surface and the presence of a greater number of oxygen functional groups on their surfaces [30]. Physical (thermal) activation involves carbonization at a temperature of 600–1000 °C to eliminate most of the volatile substances. Then, partial gasification takes place using an oxidizing gas such as CO_2_ or steam to obtain porosity and an active surface [29,31,32]. For example, CO_2_ activation of coconut shell carbons, as well as increasing the activation temperature, promoted the formation of pores and increased the volume of mesopores, while the prolonged activation time contributed to the formation of micropores and mesopores [32].

The described properties of biochars indicate that, from the point of view of their suitability for practical use, their structures are dependent on the raw materials used, the process conditions and the presence of functional groups, including oxygen groups, as well as the developed specific surface area and microporosity. Therefore, the aim of this work was to obtain biochar materials from plant biomass (corn waste and apple pomace) and to determine the changes occurring under the conditions of the pyrolysis process and physical activation, as well as to determine the physicochemical characteristics of the produced products in terms of their practical use.

## 2. Results and Discussion

**Figure 1:**
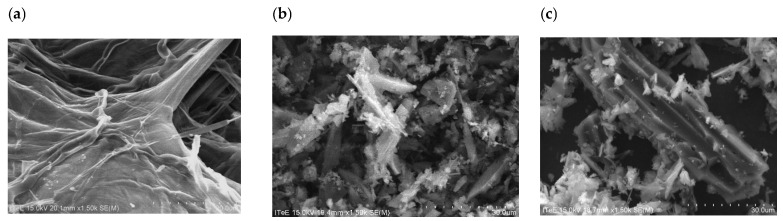
Photos of the microstructures of plant biomass from corn (**a**) before pyrolysis (K), (**b**) after pyrolysis at 700 °C (K_700) and (**c**) after pyrolysis with water vapor activation (K_700_A) (magnification ×1.50k).

**Figure 2:**
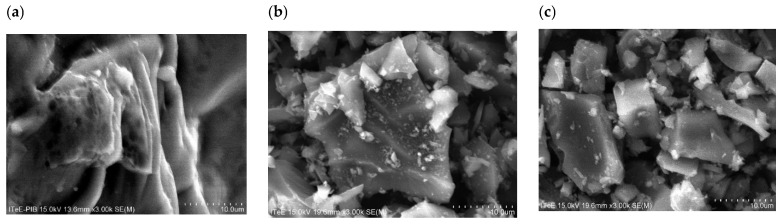
Microstructure photos of plant biomass from apples (**a**) before pyrolysis (J), (**b**) after pyrolysis at 700 °C (J_700) and (**c**) after pyrolysis with water vapor activation (J_700_A) (magnification ×3.00k).

The microstructures of corn (K) and apple (J) biomass and biochars obtained from it, before and after activation with water vapor at 700 °C, are shown in Figure 1 and Figure 2.

The photos in Figure 1 show that, as a result of the heat treatment of fibrous corn biomass, the fibers are retained, but they are broken (Figure 1b) as a result of mechanical grinding to a size of 200 µm. After activation with water steam, the microstructure of the biochar changes, resembling a “tube” shape [3,14]. Figure 1c shows depressions on the surfaces of the biochars, which may indicate the presence of pores [33]. Similar results were obtained by Sinha et al. [34], who found that the cavities in biochars indicate pores obtained as a result of pyrolysis. Similar results were observed by Singanan et al. [35] in their work, stating that the obtained SEM images illustrated the surface texture and porosity of biochar, with holes and small openings on the surface, which increased the contact area.

The photos in Figure 2 show that the apple biomass has a structure with clearly defined fibers, which is preserved during the pyrolysis process but changes when mechanically crushed to a size of 200 µm, transforming into a “flake” structure. After activation with water vapor, recesses are visible on the surfaces of the biochars (Figure 2c), which may indicate the presence of pores [33]. Similar results were observed by Qin et al. [36], who analyzed SEM images of biochars obtained from pine nut shells after steam activation. The images presented in [36] show indentations in the material, which may indicate a large number of micropores and mesopores but also macropores.

**Figure 3:**
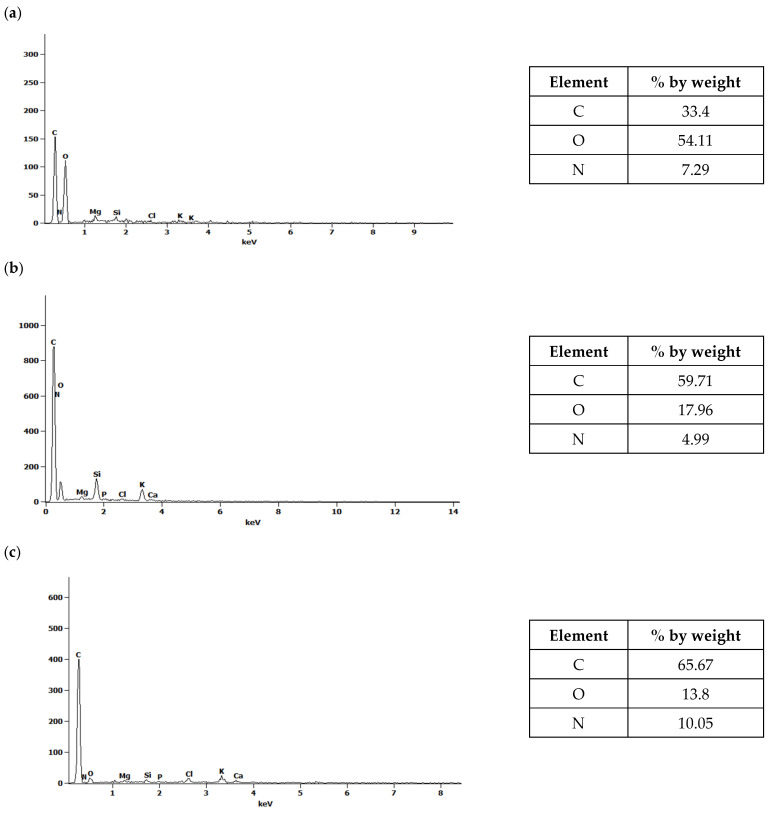
The EDS spectra of (**a**) corn biomass (K) and biochars (**b**) without activation (K_700) and (**c**) after activation with water vapor (K_700_A).

**Figure 4:**
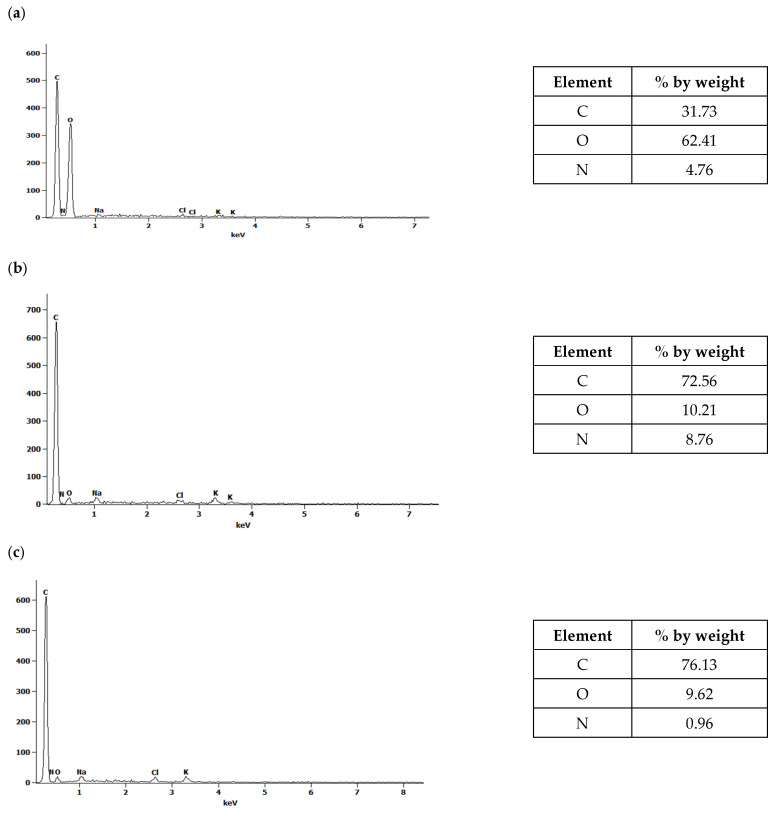
The EDS spectra of (**a**) apple biomass (J) and biochars (**b**) without activation (J_700) and (**c**) after activation with water vapor (J_700_A).

Together with microscopic examinations, an analysis of the elemental composition of the waste biomass before pyrolysis and the obtained biochars before and after water vapor activation was carried out (Figure 3 and Figure 4).

The spectra presented in Figure 3 and Figure 4 indicate the presence of carbon, oxygen and, to a lesser extent, nitrogen. The results obtained for corn biomass and the biochars obtained from it before and after activation indicate an approximately twofold relative increase in carbon in the products after pyrolysis compared to the initial product (Figure 3). In the plant biomass from apple, for biochars without and after steam activation, a 2.4-fold relative increase in carbon content was observed compared to the initial product (Figure 4). The pyrolysis process of waste biomass causes the breaking of the chemical bonds present in it and the creation of new cyclic carbon structures [37]. In addition, during the pyrolysis process, elements other than carbon are removed in the form of volatile chemical compounds [37].

**Figure 5:**
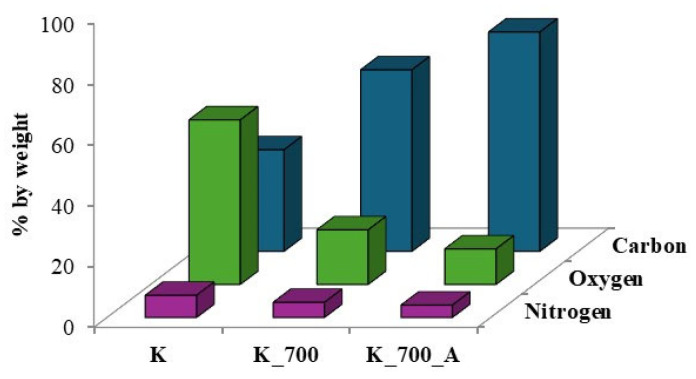
Comparison of relative percentages of carbon, oxygen and nitrogen determined in plant biomass from corn (K) and biochars before (K_700) and after water vapor activation (K_700_A).

**Figure 6:**
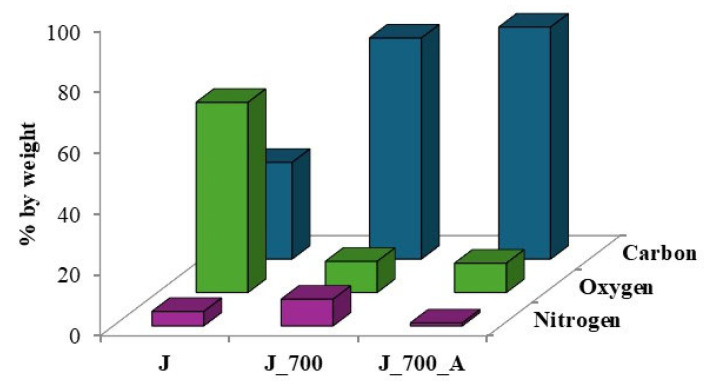
Comparison of relative percentages of carbon, oxygen and nitrogen determined in apple biomass (J) and biochars before (J_700) and after water vapor activation (J_700_A).

Figure 5 and Figure 6 compare the overall percentages of carbon, oxygen and nitrogen determined in the corn and apple biomass and the biochars before and after water vapor activation. A threefold reduction in oxygen content was observed for biochars obtained from corn before activation, as well as an approximately fourfold reduction for biochars after activation with water vapor, compared to the oxygen content in the biomass. In the case of biochars obtained from apples, before and after activation with water vapor, the oxygen loss was more than sixfold. The pyrolysis process carried out at a temperature of 700 °C results in the transformation of oxygen groups and the removal of oxygen in the gaseous products formed during pyrolysis [14], as evidenced by the oxygen losses in the biochars.

The use of the SEM/EDS technique allowed us to obtain information on the microstructures of the biochars and to identify the chemical composition changes under the influence of water vapor activation. Our research has confirmed that, depending on the biomass used and the process conditions, we can obtain biochars with varied elemental compositions. To obtain a full picture of the changes occurring in biochars under the influence of water vapor activation, further analysis of the biochars using Fourier transform infrared spectroscopy (FTIR) was required.

**Figure 7:**
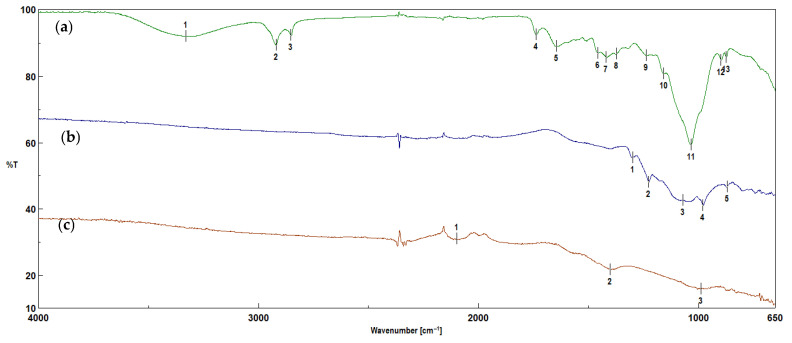
The FTIR spectra of plant biomass from corn (**a**) before pyrolysis (K), (**b**) after pyrolysis at 700 °C (K_700) and (**c**) after pyrolysis with water vapor activation (K_700_A) (descriptions of the bands are given in Table 1).

**Figure 8:**
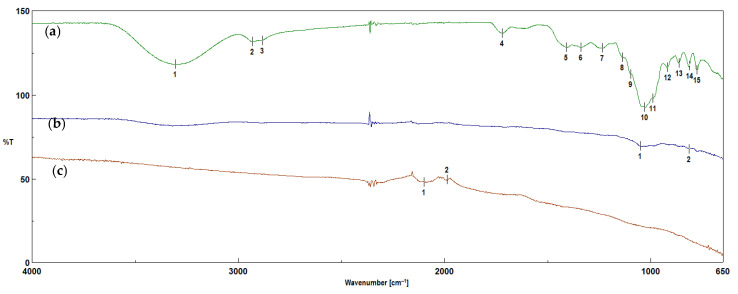
The FTIR spectra of plant biomass from apples (**a**) before pyrolysis (J), (**b**) after pyrolysis at 700 °C (J_700) and (**c**) after pyrolysis with water vapor activation (J_700_A) (descriptions of the bands are given in Table 1).

The FTIR technique was used to determine the functional groups or demonstrate their absence on the surfaces of the biochars. Figure 7 and Figure 8 show a comparison of the FTIR spectra of the biomass from corn and apples and the biochars obtained from them, before and after activation with water vapor. The characteristics of the bands obtained in corn and apple waste and the biochars before and after activation are summarized in Table 1.

The peaks appearing in the corn and apple biomass (Figure 7 and Figure 8) at wavenumbers of 3330 cm^−1^ and 3305 cm^−1^ correspond to the stretching vibrations of the –OH groups [38,39,40,41,42,43]. However, the peaks appearing at wavenumbers of 2921 cm^−1^, 2854 cm^−1^ and 2932 cm^−1^ correspond to the asymmetric and symmetric vibrations of C–H of the methylene groups [39,40,43]. These bands disappear after the pyrolysis process. The bands with wavenumbers of 1738 cm^−1^ and 1722 cm^−1^ correspond to the valence vibrations of C=O groups [35,39,40,42]. The band with a wavenumber of 1646 cm^−1^ corresponds to the valence vibrations of –C=C– groups [34,40]. The bands with wavenumbers of 1420 cm^−1^ and 1412 cm^−1^ correspond to the stretching vibrations of C–H groups [40]. The bands containing organooxygen groups, under the influence of the pyrolysis process carried out at a temperature of 700 °C, reflect the transformation and removal of oxygen in the gaseous products. In addition, hydroxyl and hydrogen radicals can also be generated—hence the absence of these groups in the biochars before and after steam activation. The spectra of biochars obtained from corn biomass show bands with wavenumbers of 1300 cm^−1^ and 1227 cm^−1^, corresponding to vibrations of the –C–O–C– [43], C–O or C–OH groups [33]. The intensity of the band at a wavenumber of 1035 cm^−1^ present in the corn biomass after the pyrolysis process is reduced and divided into two bands of 1070 cm^−1^ and 981 cm^−1^ (Table 1). The change in the intensity of the bands may indicate a decrease in the number of organooxygen groups present in the biomass compared to the obtained biochars, which indicates changes occurring in the structures of the biochars under the influence of the pyrolysis process carried out at a temperature of 700 °C. In biochars obtained from corn biomass after activation with water vapor, the band at the wavenumber of 1035 cm^−1^ disappears, leaving only the band at the wavenumber of 990 cm^−1^. The bands occurring at wavenumbers of 1138 cm^−1^, 1097 cm^−1^ and 1032 cm^−1^ in the biochars before activation are visible in the spectra in Figure 8a, whereas, after the activation process, the bands at 1138 cm^−1^ and 1097 cm^−1^ decay, creating a band with a wavenumber of 1053 cm^−1^. Similar changes occur in the intensities of the bands at wavenumbers of 1032 cm^−1^ and 1053 cm^−1^, and their complete disappearance occurs in biochars obtained from apple biomass before and after activation with water vapor. The FTIR results described by Singanan et al. [35] for biochars obtained from marigold leaves show signals at 1332 cm^−1^ and 1050 cm^−1^, indicating the –OH group belonging to phenols. Meanwhile, the peak at 757 cm^−1^ can be assigned to the deformation vibrations of the C–H bonds in the phenolic rings. The FTIR analysis performed by Suárez-García et al. [44] on biochars obtained from apples showed the presence of bands at 1110 cm^−1^, 1060 cm^−1^, 1030 cm^−1^ and 897 cm^−1^, which indicated stretching vibrations in C–O alcohols. However, the analysis of biochars conducted by Sinha et al. [34] indicated a band with a frequency of 965 cm^−1^, corresponding to C–H bending vibrations.

**Table 1:** Characteristics of the bands obtained using the FTIR technique.

Type of Biochar	Occurrence of Bands	Band [cm^−1^]	Band Characteristics	References
K	1	3330	Stretching vibrations –OH	[33,38,39,40,41,42,43,45]
	2	2921	Asymmetric and symmetric vibrations C–H	[39,40,43]
	3	2854	Asymmetric and symmetric vibrations C–H	[39,40,43]
	4	1738	Valence vibrations C=O	[33,39,40,42]
	5	1646	Valence vibrations –C=C–	[33,40]
	6	1459	Stretching vibrations C=C	[27]
	7	1420	Stretching vibrations C–H	[40]
	8	1374	Stretching vibrations C=C	[27]
	9	1239	Stretching vibrations C–O	[33]
			Stretching vibrations C–OH	[3,33]
	10	1159	Stretching vibrations C–O	[33]
	11	1035	Stretching vibrations C–O	[33]
			Stretching vibrations C–O–H	[40]
			Stretching vibrations C–C	[40]
	12	898	Plane deformation vibrations C–H in –C=C–H	[3,14,33,38,41,42]
	13	875	Stretching vibrations C–H	[33]
K_700	1	1300	Vibrations –C–O–C–	[43]
	2	1227	Stretching vibrations C–O or C–OH	[33]
	3	1070	Stretching vibrations C–O	[38]
			Stretching vibrations –C–O–C–	[39,41]
	4	981	Stretching vibrations C–O	[33,41]
			Stretching vibrations C–H	[42]
	5	871	Deformation vibrations C–H	[3,14,33]
K_700_A	1	2099	Valence vibrations C≡C	[14]
	2	1401	Plane deformation vibrations C–H in –C=C–H	[3,14,40]
	3	990	Stretching vibrations C–O	[33]
			Deformation vibrations C–H	[3,14,41,42]
J	1	3305	Stretching vibrations –OH	[33,38,39,40,41,42,43,45]
	2	2932	Asymmetric and symmetric vibrations C–H	[39,40,43]
	3	2884	Asymmetric and symmetric vibrations C–H	[39,40,43]
	4	1722	Valence vibrations C=O	[33,39,40,42]
	5	1412	Plane deformation vibrations C–H in –C=C–H	[3,14,40]
	6	1338	Stretching vibrations C=C	[27]
	7	1235	Stretching vibrations C–O or C–OH	[33]
	8	1138	Stretching vibrations C–O	[33]
	9	1097	Stretching vibrations C–O	[33]
	10	1032	Stretching vibrations –C–O–C–	[41]
	11	991	Stretching vibrations C–O	[33]
	12	918	Vibration of aromatic C-H	[42,43]
	13	865	Stretching vibrations C–H	[33]
	14	814	Stretching vibrations C–H	[33]
	15	777	Vibration of aromatic C-H	[42,43]
J_700	1	1053	Valence vibrations –C–OH	[40]
			Stretching vibrations –C–O–C–	[41]
	2	815	Vibration of aromatic C-H	[42,43]
J_700_A	1	2100	Valence vibrations C≡C	[14]
	2	1988	Asymmetric valence vibrations –C=C=C–	[14,42]

Our own studies [14] have shown that increasing the pyrolysis temperature to 700 °C leads to the disappearance of the bands indicating the presence of organooxygen structures. High-temperature pyrolysis limits the possibility of the formation of oxygen organic structures on the surfaces of biochars. The disappearance of organooxygen functional groups is also confirmed by the results obtained using the EDS technique. In Figure 5 and Figure 6, one can see the reduction in oxygen content in biochar samples obtained from corn and apple biomass before and after activation, which is removed during pyrolysis as gaseous products. After activation with water vapor, the loss of oxygen in the biochars is greater than before activation, which results in the disappearance of the organooxygen functional groups on the surfaces of the biochars, as observed in Figure 7 and Figure 8.

The use of the FTIR spectral method allowed for the identification of the chemical structures of the tested materials, as well as obtaining information on the changes occurring in the biochars as a result of the pyrolysis process and water vapor activation. To obtain a full picture of the changes occurring in biochars under the influence of water vapor activation, further analysis of the biochars using Raman spectroscopy was required.

**Figure 9:**
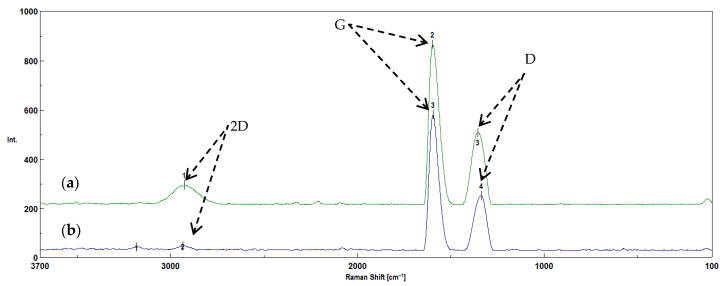
The Raman spectra of biochars obtained from plant biomass from corn (**a**) after pyrolysis at 700 °C (K_700), where 1—2927 cm^−1^, 2—1596 cm^−1^, 3—1356 cm^−1^, and (**b**) after pyrolysis with water vapor activation (K_700_A), where 1—3183 cm^−1^, 2—2936 cm^−1^, 3—1594 cm^−1^, 4—1336 cm^−1^.

**Figure 10:**
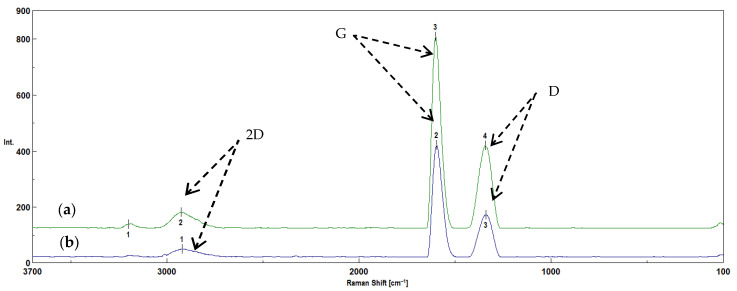
The Raman spectra of biochars obtained from plant biomass of apples (**a**) after pyrolysis with water vapor activation (J_700_A), where 1—3199 cm^−1^, 2—2927 cm^−1^, 3—1601 cm^−1^, 4—1340 cm^−1^, and (**b**) after pyrolysis at 700 °C (J_700), where 1—2922 cm^−1^, 2—1596 cm^−1^, 3—1338 cm^−1^.

Raman spectroscopy was used to evaluate the changes occurring in the structures of the biochars before and after activation, based on the analysis of the amorphous nature of the carbon structure (G bands and D bands). The bands located in the range of 1300–1400 cm^−1^ indicate a disordered structure and characterize the degree of amorphization of the carbon structure. The bands occurring at such wavenumbers are called D bands [14,44,46,47]. The bands in the range of 1500–1600 cm^−1^ are related to the graphite-like structure typical of ordered carbon materials. These bands are also called G bands. These bands occur in activated carbons and correspond to ordered graphite layers [46,47]. The bands occurring in the range of 2700–3000 cm^−1^ are called 2D bands, which occur in most carbon materials (e.g., graphite, graphene), indicating that the structure is in proximity to the graphite phase [48]. The obtained spectra of the biochars obtained from corn and apple biomass, before and after activation with water vapor, showed D, G and 2D bands (Figure 9 and Figure 10).

**Figure 11:**
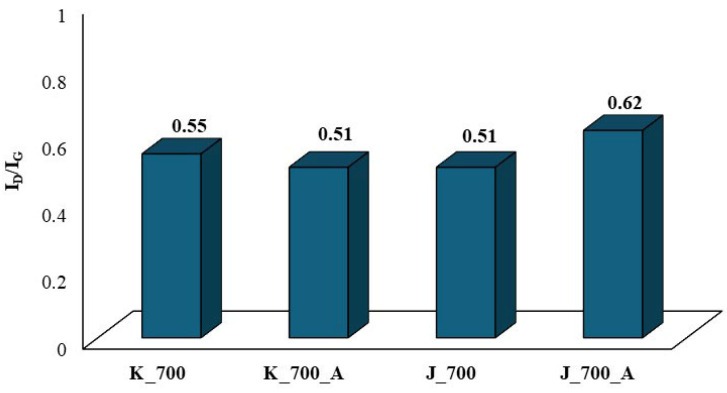
The changes in the I_D_/I_G_ coefficient determined for biochars before and after activation.

One of the most important parameters in studying the crystalline or graphite-like structure of carbon is the I_D_/I_G_ ratio (Figure 11). The surface ratio between D and G allows one to obtain information about the level of amorphousness of carbon structures (orderliness/disorderliness), which is also called the degree of graphitization [11].

The I_D_/I_G_ ratio for the biochars obtained from corn biomass before activation is 0.55, which indicates a tendency towards a disordered structure, attributed to activated carbons. After activation, the I_D_/I_G_ ratio amounts to 0.51, which indicates that the material tends to form an ordered structure, characteristic of graphite. When comparing the I_D_/I_G_ ratios for biochars obtained from corn biomass before and after water vapor activation, it is found that water vapor activation causes little change. This is confirmed by reports in the literature [49]. The small differences in the I_D_/I_G_ coefficient values for biochars obtained from corn biomass before and after activation with water vapor may also result from the decrease in the intensity of the peaks characteristic of oxygen-limiting groups obtained by the FTIR technique (Figure 7). The distinct peak at a wavenumber of 1030 cm^−1^ in the corn biomass is reduced and divided into two peaks of lower intensity (1073 cm^−1^ and 981 cm^−1^) in the biochar without activation, under the influence of the pyrolysis process and the applied temperature of 700 °C. However, in the biochar after activation with water vapor, one peak (993 cm^−1^) of a very low intensity remains. This is related to the loss of organooxygen groups, which was confirmed by tests carried out using the EDS technique (Figure 5). The loss of organooxygen groups causes changes in the structure of the biochar, as demonstrated by the I_D_/I_G_ coefficient values obtained from the analyses performed using the Raman technique.

The I_D_/I_G_ ratio for biochars obtained from apple biomass before activation is 0.51. Similarly to the biochars obtained from corn biomass, this value indicates that the material tends to have an ordered structure, attributed to graphite. However, after activation with water steam, the I_D_/I_G_ ratio is 0.62, which indicates that biochars with a disordered structure, similar to those of activated carbons, were obtained. According to Li et al. [47,48], in their studies conducted on brown coal, the I_D_/I_G_ coefficient increased only slightly or remained unchanged with an increase in the process temperature. According to the authors of [47,48], the I_D_/I_G_ ratio should decrease with an increase in the pyrolysis temperature. The conducted research shows that, in the case of the water vapor activation of apple biochars, the I_D_/I_G_ ratio increased compared to the biochars before activation. In this case, the differences result from the disappearance of the bands characteristic of oxygen-limiting groups obtained by the FTIR technique (Figure 8). The distinct peak with a wavenumber of 1026 cm^−1^ in the apple biomass decreases during the pyrolysis process in the obtained biochars without activation (1050 cm^−1^). However, after activation with water vapor, the peak at this value disappears. As in the case of the biochars obtained from corn biomass, the results obtained indicate the loss of organooxygen groups, which was confirmed by tests carried out using the EDS technique (Figure 6). The loss of these groups causes changes in the structure of the biochar, as demonstrated by the I_D_/I_G_ coefficient values obtained from the analyses performed using the Raman technique.

**Figure 12:**
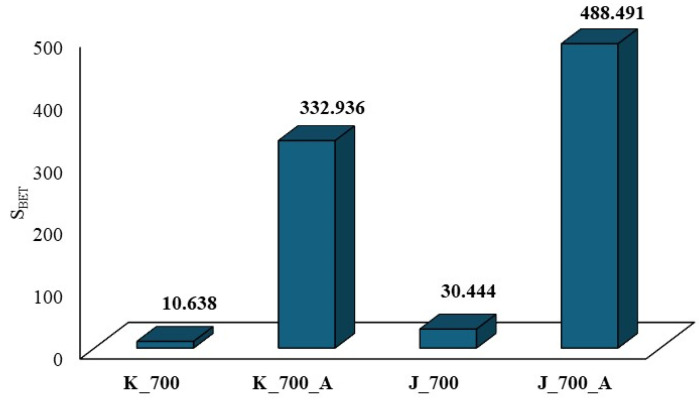
The changes in surface development for biochars obtained from plant biomass.

The tests carried out on the sorbometer allowed for the description of the surfaces. The list of parameters characterizing surface development (Figure 12), as well as the pore volume, is presented in Table 2.

The surface development of biochar products obtained from corn before activation yields a value of S_BET_ = 10.638 m^2^·g^−1^, while that obtained from apples before activation is S_BET_ = 30.444 m^2^·g^−1^ (Figure 12). After activation with water vapor, a significant increase in the surface development of the biochars obtained can be observed: for corn, S_BET_ = 332.936 m^2^·g^−1^; for apples, S_BET_ = 488.491 m^2^·g^−1^.

Activation with water vapor leads to an increase in surface development for biochars obtained from corn biomass without activation as compared to after activation, by up to 33-fold. In the case of biochars obtained from apple biomass, after activation with water vapor, this value is more than 16-fold higher compared to biochars without activation. The obtained research results confirm the literature reports. According to Devi et al. [50], the use of water vapor as an activator leads to the formation of mesopores and macropores. Activation with water vapor promotes the opening of micropores, which in turn leads to the development of the surface, which determines the sorption capacity of biochars. Similar results were observed by Qin et al. [36].

Analyzing the obtained results regarding surface development presented in Table 2 and Figure 12, it can be concluded that activation with water vapor causes changes to occur in the structures of biochars—hence the differences in the S_BET_ value or surface development for the obtained biochars. The differences in surface development for biochars after water vapor activation may result from the degree of order or disorder of the structure. Biochars activated by water vapor obtained from apple biomass show a random structure (I_D_/I_G_ ratio), based on the results obtained using the Raman technique, which tends to resemble the structure found in activated carbons. On the other hand, water vapor-activated biochars obtained from corn biomass tend to obtain an ordered structure (I_D_/I_G_ ratio) characteristic of graphite. Additionally, these biochars have organooxygen functional groups on their surfaces, which are missing in the water vapor-activated biochars obtained from apple biomass. This is confirmed by the results obtained using the FTIR technique.

## An Example of the Use of Corn Biochar for Hydrogen Sulfide Adsorption

**Figure 13:**
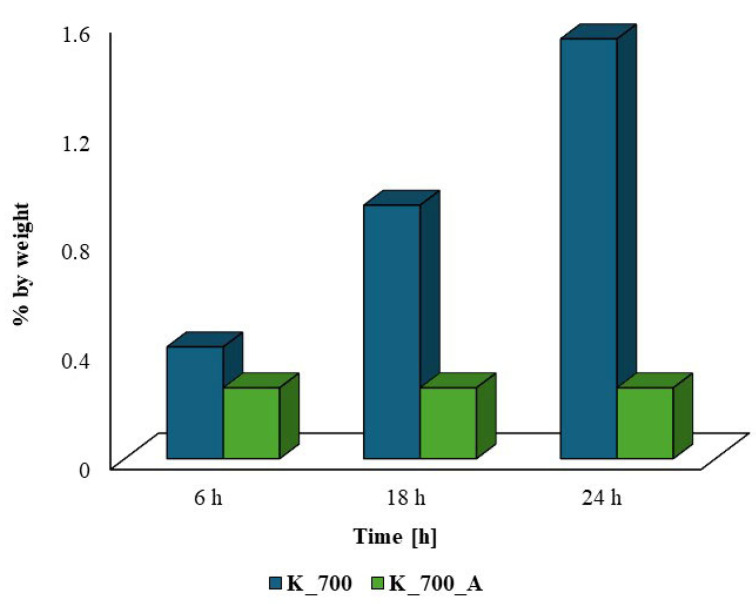
The influence of the hydrogen sulfide contact time on the sorption capacity of biochars obtained from corn before (K_700) and after activation with water vapor (K_700_A), based on EDS analysis.

The physicochemical analyses of the biochars before and after water vapor activation allowed us to obtain information on the development of the surface or S_BET_, the porosity and the presence of functional groups on their surfaces. The FTIR analyses performed showed that the biochars obtained from corn before and after activation showed the presence of functional groups capable of sorption, as compared to the biochars obtained from apple plant biomass before and after activation. The obtained corn biochars were used as hydrogen sulfide sorbents to check the influence of the pyrolysis conditions on their effectiveness. The sorption capacity of the produced biochars was successfully assessed based on the changes in sulfur content using the EDS technique. The results regarding the effects of the contact time on the sulfur content are presented in Figure 13.

Analyzing the obtained results (Figure 13 and Table 3) regarding the absorption of hydrogen sulfide using biochars, it can be observed that the best sulfur absorption capacity is demonstrated by the biochar obtained from corn waste before activation, as compared to biochars after activation. Biochars obtained from corn without activation reach their maximum sorption capacity within 24 h. However, in the case of biochars after activation with water vapor, the maximum sorption capacity is achieved after 6 h. Such differences in sulfur sorption capacity result from the ways in which the process is carried out. The pyrolysis of corn waste at 700 °C under cascade conditions leads to the disappearance of –OH groups located at wavenumbers of 3329 cm^−1^ and 3295 cm^−1^. High-temperature pyrolysis with simultaneous activation also leads to the disappearance of bands in the range of 1000–1400 cm^−1^, which indicate the presence of C=O, –C=C–, C–H, C–O and C–OH structures. It was found that conducting the pyrolysis process with simultaneous activation limited the possibility of the formation of organooxygen groups on the surfaces of the biochars, which significantly affected the sorption of hydrogen sulfide.

**Figure 14:**
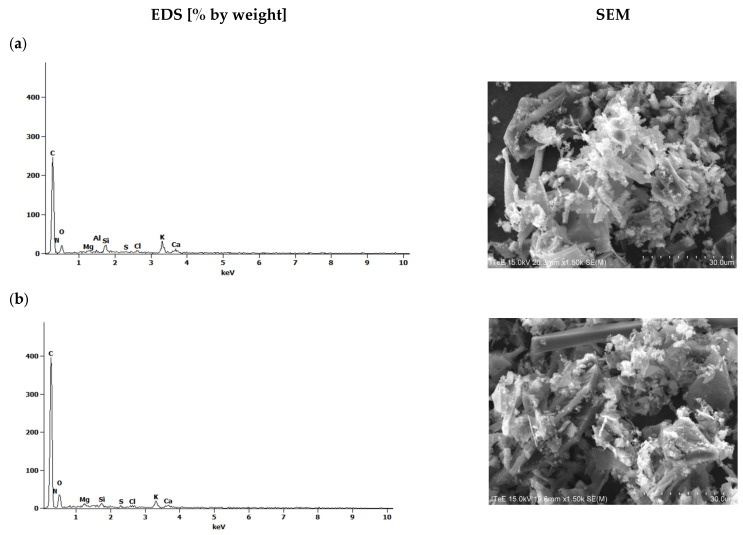

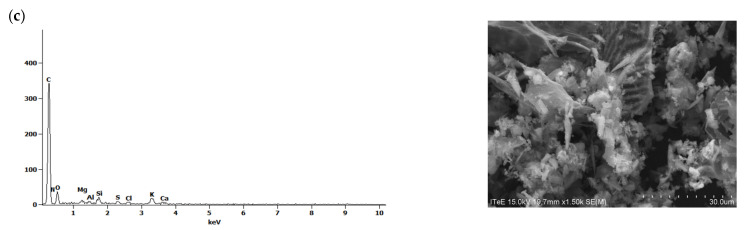
The SEM/EDS spectra recorded during hydrogen sulfide adsorption for corn biochars without activation (K_700), at adsorption times of (**a**) 6 h, (**b**) 18 h and (**c**) 24 h.

**Figure 15:**
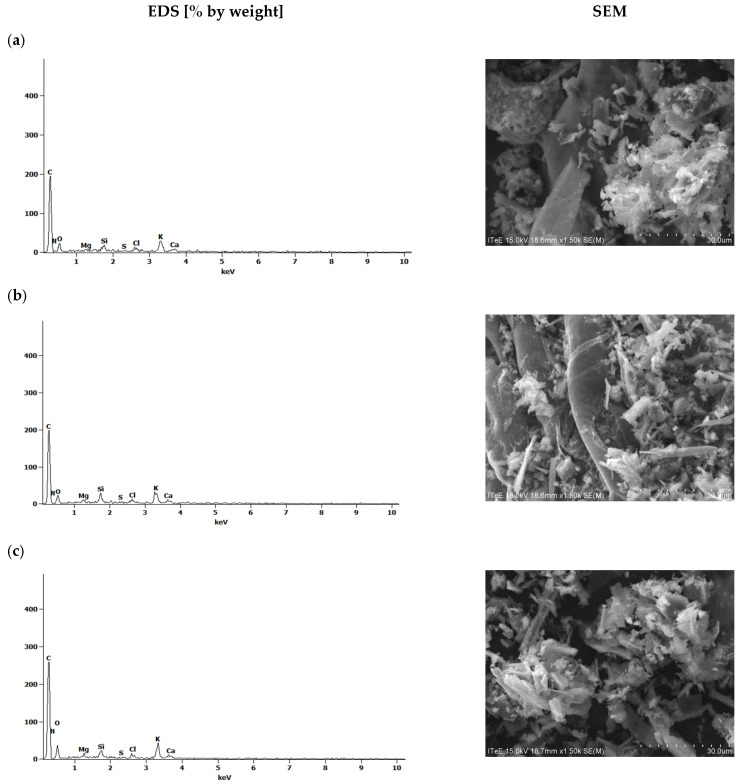
The SEM/EDS spectra recorded after the adsorption of hydrogen sulfide using corn biochars (K_700_A), at adsorption times of (**a**) 6 h, (**b**) 18 h and (**c**) 24 h.

Figure 14 and Figure 15 present the EDS results and SEM spectra of the biochars after hydrogen sulfide sorption.

The analyses carried out on the sorption capacity of biochars obtained from corn waste before and after activation with water vapor showed that the best results were obtained with biochars obtained from corn without activation with water vapor (K_700). In the case of corn biochars, the sorption capacity is due not only to the sorption surface but also to the presence of organooxygen groups on the surfaces of the biochars that exhibit a sorption capacity. Biochars without water vapor activation show more bands indicating the presence of functional groups on their surfaces than after activation with water vapor (Figure 7); hence, differences in hydrogen sulfide sorption were observed. Activation with water vapor was carried out at a temperature of 700 °C, and the contact time of the biochars with steam was 45 min. Similar results were observed by Marquez Negro et al. [51]. According to previous reports [52,53], in order to obtain the largest possible sorption surface and improve the sorption capacity of biochar products after activation with water vapor, it is recommended to carry out this process at higher temperatures.

## 3. Materials and Methods

Two types of plant biomass were selected for this study: corn waste (K) and apple waste (J)—see Table 4. The natural waste used during laboratory work was dried and crushed and had moisture content of no more than 10%, determined using the moisture analyzer method.

The analytical material consisted of biochars obtained at a laboratory stand equipped with a Czylok muffle furnace and a steam generator. During heating and cooling, the protective gas, i.e., carbon dioxide, flowed at a rate of 5.0 L/min in the chamber.

The plant biomass pyrolysis process was carried out under cascade heating conditions in a CO_2_ atmosphere in the following stages: a temperature increase from 20 °C to 200 °C in 15 min, heating at 200 °C for 15 min, a further temperature increase to 650 °C for 30 min, heating at 650 °C for 15 min, a temperature increase from 650 °C to 700 °C in 15 min and heating at 700 °C for 15 min. After this time, the heating was turned off and the sample was left in the oven for 24 h to cool down spontaneously to room temperature [23,24]. The obtained samples were marked as follows: K_700—biochar obtained from corn biomass without activation; J_700—biochar obtained from apple biomass without activation.

The pyrolysis process with simultaneous activation by water vapor activation was carried out under cascade heating conditions in a CO_2_ atmosphere in the following stages: a temperature increase from 20 °C to 200 °C in 15 min, heating at 200 °C for 15 min and a further temperature increase to 650 °C for 30 min. After reaching the temperature of 650 °C, the inert gas was turned off and water vapor activation was supplied for the final soaking period, i.e., 30 min (maximum process temperature—700 °C). After this time, the heating was turned off and the sample was left in the oven for 24 h to cool down spontaneously to room temperature [23,24]. The obtained samples were marked as follows: K_700_A—biochar obtained from corn biomass after activation; J_700_A—biochar obtained from apple biomass after activation. After the pyrolysis process with water vapor activation, the obtained biochars were crushed and sieved through 200 µm sieves.

After pyrolysis and steam activation, the biochars were subjected to tests to determine their microstructures and identify their elemental compositions using an energy-dispersive scanning electron microscope (SEM/EDS). The research was carried out on an SU-70 apparatus from Hitachi (Tokyo, Japan), equipped with an electron gun with a Schottky thermal emitter and an energy-dispersive X-ray microanalyzer EDS from ThermoScientific (Waltham, MA, USA). The apparatus settings at which the tests were carried out were as follows: voltage of 15 kV, working distance of 15 mm, beam angle of 30°, underpressure of 10^−8^ Pa (high vacuum, SE detector), magnification ×3.00k, ×1.5k. The identification of functional groups present on the surfaces of the biochars before and after activation with water vapor was performed using an FTIR spectrophotometer (ICP MS–iCAP Q, ThermoFisher Scientific, Waltham, MA, USA). The tests were performed with a Jasco 6200 FTIR spectrometer in reflectance mode (Jasco, Tokyo, Japan), using a Pike-type attachment with a diamond crystal. The apparatus settings at which the tests were carried out were as follows: spectral range of 4000–650 cm^−1^, spectral resolution of 4 cm^−1^, TGS detector, spectrum averaging from 30 scans, apodization cosine. The analysis of changes in the crystal structures of the biochars was performed using Raman spectroscopy. This was carried out using a Jasco (Tokyo, Japan) apparatus. The apparatus settings at which the tests were carried out were as follows: excitation with a laser of wavenumber 532 nm and exposure time 100 s. Spectra were recorded in the Raman shift range from 100 cm^−1^ to 3700 cm^−1^ with a resolution of 3.2 cm^−1^, a shift speed of 25.7 usecs, a normal amplitude, a single monochromator, a slit of 100 × 1000 um, an aperture of 4000 um, a 20× objective lens and laser power of 4.7 mW.

Measurements of the surface development of the biochars before and after activation with water vapor were performed using the AUTOSORB IQ sorbometer from Quantachrome (Anton Paar QuantaTec, Boynton Beach, FL, USA). The following parameters were determined during the tests: pore area determined from the BET equation (S_BET_), total pore volume (V_T_), micropore volume (V_DR_) and mesopore volume (V_mez_)—see Table 2.

## 4. Conclusions

During the pyrolysis of plant biomass from corn and apples, a number of physicochemical changes occur, leading to the production of biochars. Infrared spectroscopy (FTIR) is an important tool in identifying functional groups present on the surfaces of biochars. It also allows the determination of changes occurring in the structures of biochars as a result of the pyrolysis process and activation with water vapor. It was found that conducting the pyrolysis process at a temperature of 700 °C led to the disappearance of the organooxygen groups present in the plant biomass above 2800 cm^−1^. Moreover, after activation with water vapor, in the case of biochars obtained from apples, the disappearance of the organooxygen functional groups was observed.

The Raman technique allows the determination of the degree of order or disorder of the structure in biochars before and after activation with water vapor. The spectra obtained using the Raman technique allowed the determination of the degree of graphitization (I_D_/I_G_). The I_D_/I_G_ ratios of the biochars obtained from plant biomass from corn showed that the material tended to have a structure similar to that of activated carbon. However, after activation, this coefficient decreased the material tended to show an ordered structure, attributed to graphite. In the case of biochars obtained from apple biomass, the I_D_/I_G_ ratio was identical to that for biochars obtained from corn biomass after activation with steam. However, after activation with water steam, the I_D_/I_G_ ratio increased and the material tended to show a structure similar to that of activated carbon.

A physical and chemical sorption apparatus was used to determine the surface development (BET analysis, porosity) of the biochars before and after activation with water vapor. Activation with water vapor led to a significant increase in surface development for corn-based biochars, up to 33-fold. In the case of biochars obtained from apples, after activation with water vapor, there was a more than 16-fold increase in the surface area.

A scanning electron microscope (SEM) equipped with an X-ray microanalyzer (EDS) was used to determine the microstructures of the biochars before and after activation with water vapor. The conducted SEM image analyses showed changes in the structures of the biochars as a result of the pyrolysis process and activation with water vapor. The EDS analyses of biochars obtained from corn and apple waste before and after activation with water vapor allowed the determination of their elemental compositions. The effects of activation with water vapor in terms of increasing the carbon content in the biochars, as well as the loss of oxygen, which was removed together with the gaseous products generated during pyrolysis, were observed.

An X-ray microanalyzer (EDS) was used to determine the sorption capacity of the biochars. Biochars obtained from corn without activation with water vapor showed the best sorption capacity compared to biochars after activation with water vapor. The sorption capacity was confirmed by determining the amounts of sulfur adsorbed by the biochars. Biochars from corn without activation (K_700), despite having a smaller sorption surface compared to biochars after activation with water vapor (K_700_A), showed the best hydrogen sulfide sorption capacity. In this case, the sorption capacity was due not only to the sorption surface but also to the presence of functional groups on the biochar surface, which exhibited a sorption capacity. The increased presence of functional groups compared to biochars obtained from corn after activation with water vapor resulted in a better capacity for the sorption of sulfur, which was confirmed by EDS tests.

## 5. Patents

The research described above is the subject of a patent application entitled “Method of thermal production of modified biochars used as metal sorbents from aqueous solutions and biochar sorbents obtained by this method” by Kaźmierczak B., Radulski P., Kaczmarczyk A., Drabik J., Molenda J., Rogoś E., patent application number P.450650, which was filed on 19 December 2024 [in Polish] [54]. The research described above is also the subject of a patent entitled “Biocarbon sorbents of heavy metals, in particular chromium (III), obtained from waste plant biomass and the method of obtaining them”, Molenda J. Kaźmierczak B., Swat M., Rogoś E., Wolszczak M., patent number Pat. 242008, 2020 [in Polish] [55].

## Figures and Tables

**Table 2:** Surface development parameters of biochars obtained from plant biomass.

Type of Biochar	S_BET_ [m^2^·g^−1^]	V_T_ [cm^3^·g^−1^]	V_DR_ [cm^3^·g^−1^]	V_mez_ [cm^3^·g^−1^]
K_700	10.638	0.013	0.008	0.005
K_700_A	332.936	0.206	0.192	0.013
J_700	30.444	0.026	0.020	0.006
J_700_A	488.491	0.284	0.277	0.007

**Table 3:** Characteristics of biochars obtained from corn (K) after hydrogen sulfide sorption, determined by the EDS method.

Biochar	Sorption Time	EDS [% by Weight]
	[h]	Sulfur S
K_700	6	0.41
	18	0.93
	24	1.54
K_700_A	6	0.26
	18	0.26
	24	0.26

**Table 4:** Characterization of plant biomass before pyrolysis.

Sample	EDS [% by Weight]										
	C	N	O	K	Ca	Si	Cl	Mg	Px	Na	S
K	33.40	7.29	54.11	0.60	0.00	1.71	0.63	2.26	0.00	0.00	0.00
J	31.73	4.76	62.41	0.36	0.00	0.00	0.35	0.00	0.00	0.36	0.00

## Data Availability

The data presented in this study are available upon request from the corresponding author.

## References

[1] Debevc S., Weldekidan H., Snowdon M.R., Vivekanandhan S., Wood D.F., Misra M., Mohanty A.K. (2022). Valorization of almand Shell biomass to biocarbon materials: Influence of pyro lysis temperature on their physicochemical properties and electric al conductivity. Carbon Trends.

[2] (2018). The Closedloop—Open Opportunities.

[3] Kaźmierczak B., Molenda J., Swat M. (2021). The adsorption of chromium (III) ions from water solutions on biocarbons obtained from plant waste. Environ. Technol. Innov..

[4] Directive (EU) 2018/2001 of the European Parliament and of the Council of 11 December 2018 on the Promotion of the Use of Energy from Renewable Sources (Recast). https://eur-lex.europa.eu/eli/dir/2018/2001/oj/eng.

[5] Act of 7 June 2018 Amending the Act on Renewable Energy Sources and Certain Other Acts. https://isap.sejm.gov.pl/isap.nsf/DocDetails.xsp?id=WDU20180001276.

[6] Suárez L., Benavente-Ferraces I., Plaza C., Pascual-Teresa S., Suárez-Ruiz I., Centeno T.A. (2020). Hydrothermal carbonization as a sustainable strategy for integraf valorisation of apple waste. Bioresour. Technol..

[7] Rydzewska–Włodarczyk M., Drozłowska E., Sobieraj M. (2018). Calculation of production costs of products made from production residues on the example of fruit and vegetable processing. Optimum. Econ..

[8] Mirowski T., Mokrzycki E., Uliasz–Bocheńczyk A. (2018). Energy Use of Biomas.

[9] Lewandowski W.M., Radziemska E., Ryms M., Ostrowski P. (2010). Modern methods of thermochemical conversion of biomass into gaseous, liquid and solid fuels. Proc. ECOpole.

[10] Figueiredo J.L., Pereira M.F.R., Freitas M.M.A., Orfao J.J.M. (1999). Modification of the surface chemistry of activated carbons. Carbon.

[11] Kaźmierczak B., Drabik J., Wolszczak M., Radulski P., Molenda J., Hubicki Z. (2024). The influence of pyrolysis temperature on qualitative changes in carbon materials obtained from tea bio-waste. The Monograph Science and Industry—Challenges and Opportunities.

[12] Novak J.M., Lima I., Xing B., Gaskin J.W., Steiner C., Das K.C., Ahmenda M., Rehrah D., Watts D.W., Busscher W.J. (2009). Characterization of designer biochor produced at different temperatures and their effects on loamysand. Ann. Environ. Sci..

[13] Kong F., Xie Y., Xia C., Huang H., Liang D., Qiu Y., Zhang Q., Liu X., Shao H., Meng Z. (2023). Removal of hydrogen sulfide by layered double hydroxide loaded biochar in dynamic adsorption experiment. Surf. Interfaces.

[14] Molenda J., Kaźmierczak B. (2019). The use of spectralmethods to identify the microstructure and chemical structure of biocarbons used in the processes of the exploitation of technological liquids. J. Mach. Constr. Maint..

[15] Dąbek L. (2015). Application of sorption and advanced oxidation to remove phenols and their derivatives from water solutions. Middle Pomeranian Sci. Soc. Environ. Prot. Annu. Set Environ. Prot..

[16] Tan C., Zeyu Z., Sai X., Hongtao W., Wenjing L. (2015). Adsorption behavior comparison of trivalent and hexavalen tchromium on biochor derived from municipal sludge. Bioresour. Technol..

[17] Tyagi U. (2022). Enhanced adsorption of metal ions on to *Vetiveria zizanioides* biochar via batch and fixe bed studies. Bioresour. Technol..

[18] Hashem M.A., Mim M.W., Noshin N., Maoya M. (2024). Chromium adsorption capacity from tannery wastewater on thermally activated adsorbent derived from kitchen waste biomass. Clean. Water.

[19] Reczek L., Michel M.M., Świątkowski A., Tytkowska M., Trykowski G. (2017). Studies on Lead(II) Sorption from Aqueous Solutions on Fly Ash from Combustion/Co-combustion of Biomass. Eng. Prot. Environ..

[20] Kobyłka A. (2016). Application of activated carbonad sorption in various technological systems of sewage treatment plants. Tech. Issues.

[21] Kaleta J., Kida M., Koszelnik P., Papciak D., Puszkarewicz A., Tchórzewska-Cieślak B. (2017). The use of activated carbons for removing organic matter from groundwater. Arch. Environ. Prot..

[22] Khazaei I., Aliabadi M., Hamed Mosavian H.T. (2011). Use of Agricultural Waste for Removal of Cr(VI) from Aqueous Solution. Iran. J. Chem. Eng..

[23] Rodrigo P.M., Kommalapati R.R. (2025). Synergistic effect of magnetic magnetite and greigite nanoparticles dispersed pine wood biochar for aqueous lead(II) and cadmium (II) adsorption. Clean. Water.

[24] Yuan X., Zhang X., Lv H., Xu Y., Bai T. (2022). Co-Pyrolysis of Cotton Stalks and Low-Density Polyethylene to Synthesize Biochar and Its Application in Pb(II) Removal. Molecules.

[25] Chen Q., Zhang T.C., Ouyang L., Yuan S. (2022). Single-Step Hydrothermal Synthesis of Biochar from H_3_PO_4_-Activated Lettuce Waste for Efficient Adsorption of Cd(II) in Aqueous Solution. Molecules.

[26] Glaser B., Lehmann J., Zech W. (2002). Ameliorating Physical and Chemical Properties of Highly Weathered Soils in the Tropics with Charcoal: A Review. Biol. Fertil. Soils.

[27] Jindo K., Mizumoto H., Sawada Y., Sanchez-Monedero M.A., Sonoki T. (2014). Physical and chemical characterization of biochars derived from different agriculturalr esidues. Biogeosciences.

[28] Ahmed M.J. (2017). Adsorption of quinolone, tetracycline, and penicillin antibiotics from aqueous solution Rusing activated carbons: Review. Environ. Toxicol. Pharmacol..

[29] Wei Q., Chen Z., Cheng Y., Wang X., Yang X., Wang Z. (2019). Preparation and electrochemical performance of Orange peel based activated carbons activated by different activators. Colloids Surf. A.

[30] Panwar N.L., Pawar A. (2022). Influence of activation conditions on the physicochemicalproperties of activated biochar: A review. Biomass Convers. Biorefinery.

[31] Mohan D., Pittman C.U. (2006). Activated carbons and low cost adsorbents for remediation of tri– and hexavalent chromium from water. J. Hazard. Mater. B.

[32] Guo S., Peng J., Li W., Yang K., Zhang L., Zhang S., Xia H. (2009). Effects of CO_2_ activation on porous structures of coconut shell—Based activated carbons. Appl. Surf. Sci..

[33] Perumal R.S., Muralidharan B. (2024). Valorization of *Ricinus communis* outer shell biomass to biochar: Impact of thermal decomposition temperature on physicochemical properties and EMI shielding performance. Results Eng..

[34] Sinha R., Kumar S., Singh R.K. (2013). Production of biofuel and biochar by thermal pyrolysis of linseed seed. Biomass Convers. Biorefinery.

[35] Singanan M., Peters E. (2013). Removal of toxic heavy metals from synthetic wastewater using a novel biocarbon technology. J. Environ. Chem. Eng..

[36] Qin L., Hou Z., Lu S., Liu S., Liu Z., Jiang E. (2019). Porous Carbon derived from Pine Nut Shell prepared by Steam Activation for Supercapacitor Electrode Material. Int. J. Electrochem. Sci..

[37] Collard F.X., Blin J. (2014). A review on pyrolysis of biomass constituents: Mechanisms and composition of the products obtained from the conversion of cellulose, hemicelluloses and lignin. Renew. Sustain. Energy Rev..

[38] Huang S., Liang Q., Geng J., Luo H., Wei Q. (2019). Sulfurized Biochar Prepared by simplified technic with superior adsorption property towards aqueous Hg(II) and adsorption mechanisms. Mater. Chem. Phys..

[39] Misnon I.I., Zain N.K.M., Aziz R.A., Vidyadharan B., Jose R. (2015). Electrochemical properties of carbon from oil palm kernel shell for high performance super capacitors. Electrochim. Acta.

[40] Bhagat T.S., Pancharathi R.K. (2025). Performance, microstructure and carbons equestration potential of agro biochar based cement mortars. Cem. Concr. Compos..

[41] Sandhya Rani N., Manjunatha M.S., Sannappa J., Demappa T. (2018). Studies of Hydrogen Bond between HPMC Doped CdCl_2_ Polymerusing FTIR Technique. Mater. Today Proc..

[42] Geetha T., Smitha J.K., Sebastian M., Litty M.I., Joseph B., Joseph J., Nisha T.S. (2024). Synthesis and characterization of nano iron oxide biochor composite for efficient removal of crystal violet from water. Heliyon.

[43] Xu Z., Sun Z., Zhou Y., Chen W., Zhang T., Huang Y., Zhang D. (2019). Insights into the pyro lysis behavior and adsorption properties of activated carbon from waste cottontextiles by FeCl_3_-activation. Colloids Surf. A.

[44] Suárez-García F., Martínez-Alonso A., Tascón J.M.D. (2002). A comparative study of the thermal decomposition of apple pulp in the absence and presence of phosphoric acid. Polym. Degrad. Stab..

[45] Know C.W., Tae S., Mandal S. (2025). Comparative Analysis of CO_2_ Adsorption Performance of Bamboo and Orange Peel Biochars. Molecules.

[46] Grodecki K. (2013). Raman spectroscopy of graphene. Electron. Mater..

[47] Li X., Hayashi J., Li C.-Z. (2006). Volatilisation and catalytic effects of alkali and alkaline earth metallic species during the pyrolysis and gasification of Victorian Brown coal. Part VII. Raman spectroscopic study on the changes in char structure during the catalytic gasification in air. Fuel.

[48] Li X., Hayashi J., Li C.-Z. (2006). FT–Raman spectroscopic study of the evolution of char structure during the pyrolysis of a Victorian brown coal. Fuel.

[49] Grimm A., Simõesdos Reis G., Khokarale S.G., Ekman S., Lima E.C., Xiong S., Hultberg M. (2023). Shiitake spent mushroom substrate as a sustainable feedstock for developing highly efficient nitrogen-doped biochars for treatment of dye-contaminated water. J. Water Process Eng..

[50] Devi N.S., Hariram M., Vivekanandhan S. (2021). Modification techniques to improve the capacitive performance of biocarbon materials. J. Energy Storage.

[51] Marquez Negro A., Marti V., Sanchez-Hervas J.M., Ortiz I. (2025). Development of Pistachio Shell-Based Bioadsorbents Through Pyrolysis for CO_2_Capture and H_2_S Removal. Molecules.

[52] Wu F.C., Tseng R.L., Hu C.C., Wang C.C. (2004). Physical and electrochemical characterization of activated carbons prepared from firwoods for super capacitors. J. Power Sources.

[53] Qu W.H., Xu Y.Y., Lu A.H., Zhang X.Q., Li W.C. (2015). Converting biowastecorn dobre sidue into high value addend Poros carbon for super capacitor electrodes. Bioresour. Technol. Y.

[54] Kaźmierczak B., Radulski P., Kaczmarczyk A., Drabik J., Molenda J., Rogoś E. (2024). Method of Thermal Production of Modified Biochars Used as Metal Sorbents from Water Solutions and Biochar Sorbents Obtained by This Method. Patent.

[55] Molenda J., Kaźmierczak B., Swat M., Rogoś E., Wolszczak M. (2020). Biocarbon Sorbents of Heavy Metals, in Particular Chromium (III), Obtained from Waste Plant Biomass and the Method of Obtaining Them. Patent.

